# Tyroxine Hydroxylase-Positive Neuronal Cell Population is Increased by Temporal Dioxin Exposure at Early Stage of Differentiation from Human Embryonic Stem Cells

**DOI:** 10.3390/ijms20112687

**Published:** 2019-05-31

**Authors:** Sailendra Nath Sarma, Reiko Nagano, Seiichiroh Ohsako

**Affiliations:** 1 Laboratory of Environmental Health Sciences, Center for Disease Biology and Integrative Medicine, Graduate School of Medicine, The University of Tokyo, 7-3-1 Hongo, Bunkyo-ku, Tokyo 113-8655, Japan; sailendra.sarma@canada.ca; 2 Earth-Life Science Institute, Tokyo Institute of Technology, 2-12-1 Ookayama, Meguro-ku, Tokyo 152-8550, Japan; reiko.nagano@elsi.jp

**Keywords:** human, dioxin, TCDD, embryonic stem cell, embryoid body, neural differentiation, thyroxine hydroxylase, aryl-hydrocarbon receptor

## Abstract

Background: The neurological effects of short-term dioxin exposure during the fetal period is an important health risk in humans. Here, we investigated the effects of dioxin on neural differentiation using human embryonic stem cells (hESCs) to evaluate human susceptibility to dioxin. Methods: Using an enzymatic bulk passage, neural differentiation from human ESCs was carried out. 2,3,7,8-Tetrachlorodibenzo-*p*-dioxin (TCDD) was added to various stages of culture. The expression levels of the neuronal markers microtubule-associated protein 2 (MAP2) and thyroxine hydroxylase (TH) were measured by RT-qPCR and image analysis of immunostaining. Results: Although early-stage neuronal cells are quite resistant to TCDD, the numbers of neural rosettes and increases in mRNA expression levels and the number of cells positive for MAP2 and TH were significant by temporal exposure at embryoid body stage (Day9-exposure group). In contrast, the TCDD exposures against ESCs (Day0-exposure group) and differentiated neural cells (Day35-exposure group) were not affected at all. The increment was similarly observed by continuous exposure of TCDD from Day9 through Day60. Conclusions: These results indicated that dioxin exposure during the early stage of differentiation from hESCs increases the contents of neuronal cells, especially TH-positive neuronal cells. Regulations of aryl hydrocarbon receptor (AHR) signaling in an early stage of embryogenesis should be investigated extensively to understand the mechanism underlying the increase in neuronal cell populations and to apply the knowledge to regenerative medicine.

## 1. Introduction

Dioxin and related substances are ubiquitous environmental pollutants causing a wide variety of pathological alterations that affect human health owing to a diverse range of toxic effects [1]. The most toxic congener, 2,3,7,8-tetrachlorodibenzo-*p*-dioxin (TCDD), is produced from the low-temperature burning of chlorine-containing organic compounds, such as waste dump fires [2]. Dioxin exposure continues to represent a significant global public health issue with potentially greater adverse effects on populations exposed during development because the effects can be longer lasting and may occur at low doses [3,4]. Epidemiological studies have suggested that children accidentally exposed to dioxins exhibit delayed motor development and a tendency to hyperactivity [5]. Neurobehavioral abnormalities caused by dioxin exposure are associated with both cognitive and locomotor systems in humans [6].

The aryl hydrocarbon receptor (AHR) is well known as a dioxin receptor. Since no target molecule with higher affinity for TCDD than Ahr has been reported, all pathological changes caused by TCDD are considered to be mediated by Ahr signaling [7,8]. Ahr-mediated signaling is suspected to target the central nervous system during the early developmental stage in laboratory mouse models, as shown by numerous descriptive reports on, for example, abnormal behavior, recognition disorder, or abnormal neuronal cell migration associated with TCDD exposure [9,10,11,12,13]. Unfortunately, it is often found that experimental results of TCDD toxicity obtained from laboratory animals do not represent the real situation that occurs in humans [14,15,16,17,18]. Large differences in TCDD toxicity have been known between species, especially lethal doses that may vary from 0.6 µg/kg to 5050 µg/kg between guinea pig and hamster [19]. Due to the large difference in toxicity, in vitro studies by using human stem cells as models are important for determining TCDD toxicity against central nervous system development and neuronal cell differentiation during an early stage of the fetus.

Before the establishment of induced pluripotent stem cells (iPSCs) [20,21], human embryonic stem cells (hESCs) had been expected as powerful materials for drug discovery as well as regenerative medicine [22,23,24,25]. However, hESCs are difficult to establish due to the small chances in recruiting donors and because the use of human embryos has raised ethical issues. Moreover, owing to the intactness of their genome sequence, hESCs are believed to more faithfully mimic normal and healthy human embryogenesis and the kinetics of the epigenetic landscape than iPSCs, which are modified genetically [26]. Therefore, the use of hESCs in embryonic stem cell tests (ESTs) can provide more reliable toxicological data than that of iPSCs. hESCs have toxicological applications, because their derivatives exhibit normal, nonpathological properties, which are not found in models based on cancer cells or animal stem cells [27,28]. Although ESTs using human ES/iPS cells are an innovation in verifying the effects of numerous chemical substances, there are still many unresolved issues, because stable and objective numerical values are required for toxicity evaluation [29].

In this study, we observed the developmental effects of TCDD on the neural differentiation system using hESCs. An enzymatic bulk-passage culture is able to monitor the ratio of several cell types undergoing neural differentiation at the same time as cell viability. Dopamine-producing neurons play a central role in various brain functions, such as motor control, cognition, memory processing, and emotion [30,31]. We used a bulk-passage culture protocol and evaluated TCDD effects on differentiation, focusing especially on neuronal cells positive for thyroxine hydroxylase (TH), which is a rate-limiting enzyme of dopamine synthesis [32]. Moreover, we tried transgene experiments for establishing pluripotent stem cell battery through simple live-imaging.

## 2. Results

### 2.1. TCDD Exposure at EB Stage Increased Visible Neural Rosette Number

**Figure 1:**
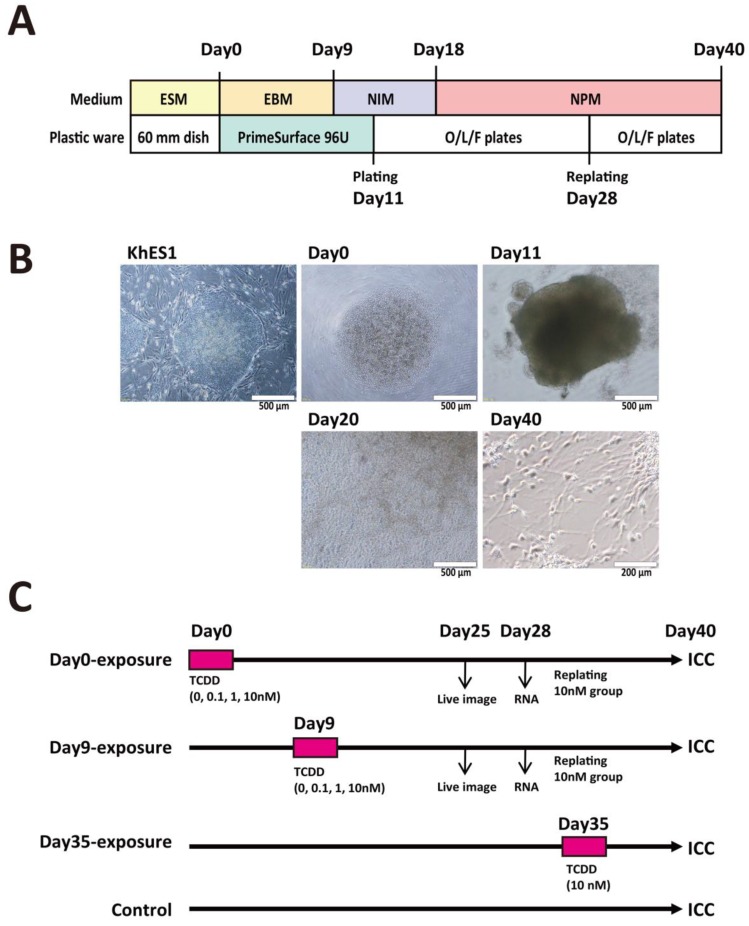
Schematic diagram of the bulk-passage system for neural differentiation using human embryonic stem cells (ESCs) and the experimental schedule for 2,3,7,8-tetrachlorodibenzo-*p*-dioxin (TCDD) exposures. (**A**) Culture schedule, media, and plastic ware in the protocol. hESCs were sub-cultured on mouse embryonic fibroblasts (MEFs) as feeder cells in embryonic stem cell medium (ESM). Embryoid bodies (EBs) were formed in PrimeSurface 96U plates with embryoid body medium (EBM) containing the ROCK inhibitor Y-27632 as described in Materials and Methods. The growing EBs were cultured for 2 additional days in neuronal induction medium (NIM) to promote neural differentiation. Then, EBs were replated onto ornithine/laminin/fibronectin (O/L/F)-coated plates. On Day18, the medium was changed with neural proliferation medium (NPM). On Day28, all cells in one well were detached with Accutase and replated onto new O/L/F-coated plates. (**B**) Typical features of cells and cell masses at various stages. Upper left, KhES1 colony. Upper middle, hESCs immediately after plating onto PrimeSurface 96U. Upper right, EB on Day11. Lower middle, cell masses on Day20. Lower right, neuronal cells on Day40. (**C**) Experimental schedule for examining the effects of TCDD exposure of cells at different stages. On Day0 immediately after plating single KhES1 cells on PrimeSurface 96U, TCDD (0, 0.1, 1, 10 nM) was added to EBM and the cells were incubated for 24 h (Day0-exposure group). On Day9 at the EB stage, TCDD (0, 0.1, 1, 10 nM) was added and the EBs were incubated for 24 h (Day9-exposure group). On Day35, mature neuronal cells were exposed to TCDD (10 nM) for 24 h (Day35-exposure group). Total RNAs were collected to measure specific mRNA on Day28. Immunocytochemistry (ICC) was performed on Day40.

**Figure 2:**
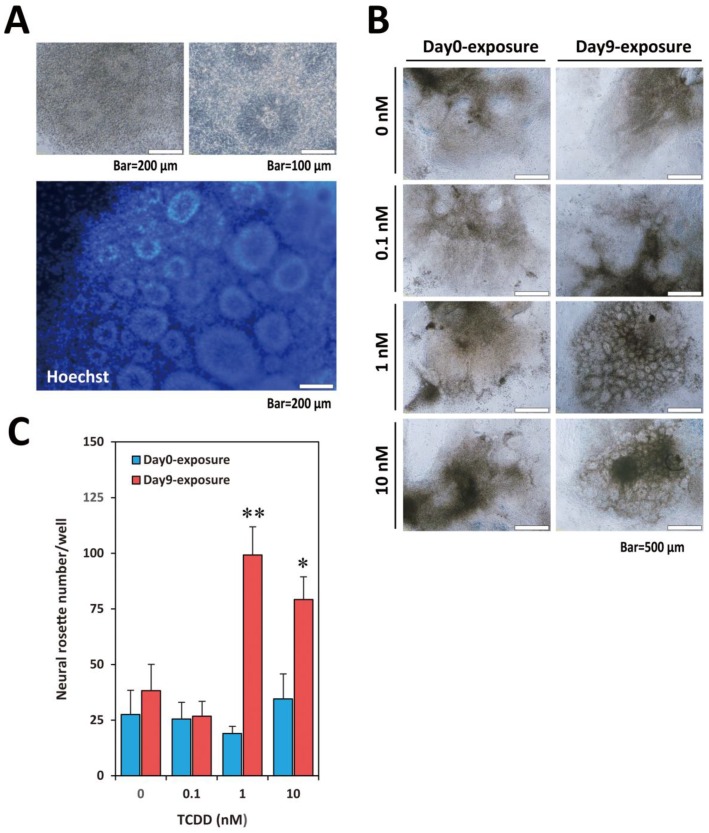
Effects of TCDD exposure on the neural rosette appearance. (**A**) Representative neural rosette. Lower panel shows results for Hoechst staining. (**B**) Bright-field image of differentiating neural cells on Day25. Compared with Day0-exposure groups, numbers of neural rosettes were remarkably increased in Day9-exposure groups in the dishes with 1 nM and 10 nM TCDD. (**C**) Neural rosette number. The total number of visible and countable rosettes was counted in one well (*N* = 4). One-way ANOVA was carried out followed by Dunnett’s test as the post hoc (*, *p* < 0.05; **, *p* < 0.01).

Neuronal cell differentiation by bulk-passage protocol is shown in Figure 1A and typical bright-field images of hES colony, embryoid body (EB), and matured neuronal cells are presented in Figure 1B. We exposed KhES1 cells (on Day0) and EBs (on Day9) to TCDD at various concentrations (0.1, 1, 10 nM) for 24 h and examined the effects of TCDD on neural differentiation (Figure 1C). During continuous culture after the withdrawal of TCDD, neural rosettes, which are ring-like structures indicating the sprouting neuronal progenitors, were observed seven days after changing neural proliferation medium (NPM) (Figure 2A). The number of countable neural rosettes observed in one well on Day25 was significantly larger in the Day9-exposure group than in the Day0-exposure group (Figure 2B,C). The statistical significance of the increase in the neural rosette number was detected at 1 nM and 10 nM (Figure 2C).

### 2.2. TCDD Exposure at EB Stage Increased Expression Levels of Neuronal Marker mRNAs

**Figure 3:**
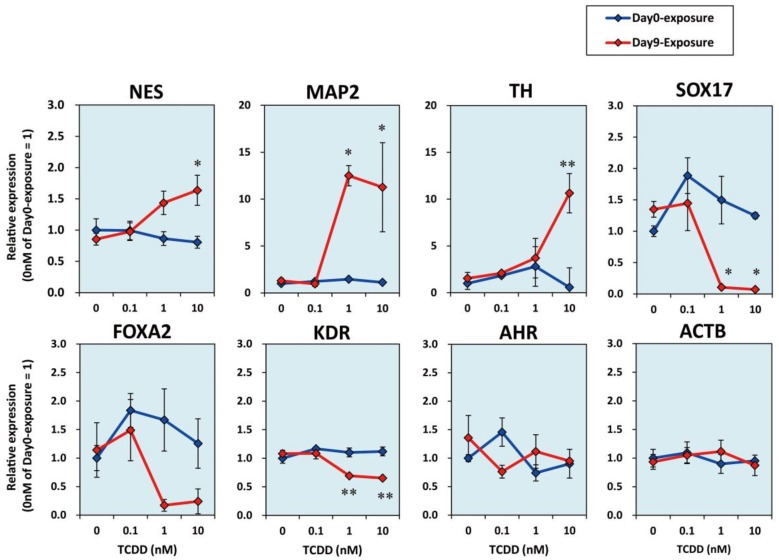
Effects of TCDD exposure on the mRNA expression levels of neural and germ layer markers. On a 24-well plate, all cells were lysed on Day28 of culture (Figure 1C). Blue line, Day0-exposure groups; Red line, Day9-exposure group. The *NES, MAP2, TH, SOX17, FOXA2, KDR, AHR*, and *ACTB* mRNAs were subjected to RT-qPCR analyses. Data are expressed as mean ± SE (*N* = 3). One-way ANOVA was carried out followed by Dunnett’s test as post hoc (*, *p* < 0.05; **, *p* < 0.01).

We then checked the mRNA expression levels of germ layer markers on Day28. The mRNA expression levels of the neuronal marker genes nestin (*NES*) [33], microtubule-associated protein 2 (*MAP2*) [34], and tyrosine hydroxylase (*TH*) [35] increased in the Day9-exposure group in a dose-dependent manner (Figure 3). In contrast, SRY-box 17 (*SOX17*) and forkhead box A2 (*FOXA2*) as the endoderm markers [36,37] and the kinase insert domain receptor (*KDR*) as the mesoderm marker [38] showed dose-dependent decreases in their relative mRNA expression levels in the Day9-exposure group. The Day0-exposure group did not show any significant difference in the expression levels of these markers with the increase in the dose of TCDD (Figure 3).

### 2.3. TCDD Exposure at EB Stage Increased Neuronal Cell Population

**Figure 4:**
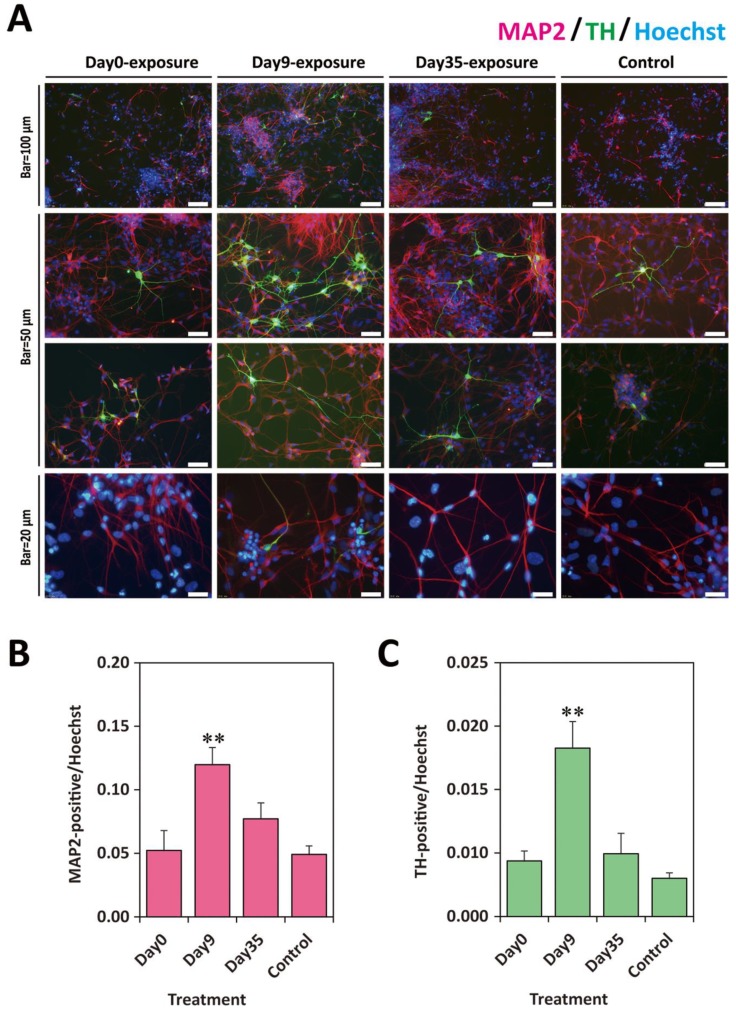
ICC analysis on Day40 of cells exposed to TCDD at different stages of differentiation. (**A**) Typical microscopy images of neural cells derived from KhES1 on Day40. Day0-exposure group, the cells exposed to 10 nM TCDD from Day0 for 24 h; Day9-exposure group, the cells exposed to 10 nM TCDD from Day9 for 24 h; Day35-exposure group, the cells exposed to 10 nM TCDD from Day35 for 24 h; Control, the cells differentiated following the same protocol without treatment. Red, MAP2-immnostaining; Green, TH-immunostaining; Blue, nucleus stained with Hoechst 33342. (**B**) Ratio of MAP2-positive cells in the culture. The *y*-axis indicates the number ratio of MAP2-positive cells per Hoechst counts. (**C**) Ratio of TH-positive cells in culture. The *y*-axis indicates the numbers ratio of TH-positive cells per Hoechst counts. Data from four images per group are expressed as mean ± SE. One-way ANOVA was carried out followed by Dunnett’s test as post hoc (**, *p* < 0.01).

On Day28, the differentiated cells exposed to 10 nM TCDD were replated onto other O/L/F-coated plates and cultured for an additional 12 days (Figure 1C). On Day40, the cells were fixed and immunostained with anti-MAP2 and anti-TH antibodies (Figure 4). The images of the cells of the Day9-exposure group presented a higher ratio of MAP2-positive cells per Hoechst-positive nuclei than those of the Day0- and Day35-exposure groups. Moreover, TH-positive cells were observed in all culture wells, but the ratio of TH-positive cells was higher in the Day9-exposure group (Figure 4A). Image analysis revealed that the ratio of MAP2-positive cells was significantly increased in the Day9-exposure group (Figure 4B). The ratio of TH-positive cells was also significantly increased in the Day9-exposure group (Figure 4C).

### 2.4. Rat Th-EGFP Trangene Did Not Work in the Human ESC-Derivatives

A construct of the plasmid prTH-EGFP-RAG-DsRed-IRESneo was designed as shown in Figure S1A (Supplementary Materials), which contains approximately 10-kb rat-*Th* promoter connected with EGFP and a rat β-actin promoter connected with DsRed. This plasmid was transfected into human hepatoma HepG2, rat pheochromocytoma PC12, and human neuroblastoma SK-N-SH. No EGFP fluorescence was detected in HepG2, but DsRed fluorescence was clearly shown (Figure S1B, Supplementary Materials), indicating that the rat β-actin promoter-DsRed cassette worked well. The EGFP- and DsRed-double-positive neuronal cells were detected by transfection of prTH-EGFP-RAG-DsRed-IRESneo into NGF-stimulated PC12 and SK-N-SH cells (Figure S1B, Supplementary Materials), suggesting that the construct is useful for monitoring the differentiation of human neuronal cells expressing the *TH* gene. The linearized construct was transfected into KhES1 cells and several clones were selected on the basis of the presence of G418. One stable ESC line was named KhES1rTHEGFP. Then, EB formation and neural differentiation cultures were carried out by our standard bulk-passage culture protocol (Figure S2A, Supplementary Materials). However, EGFP-positive cells with neural dendrite processes were rarely observed. A few EGFP-positive cells having neuron-like processes were observed among all the neuronal cells growing in a culture well (Figure S2B, Supplementary Materials). Additionally, no DsRed fluorescence was clearly detected. However, a number of TH-positive neuronal cells were observed by ICC using anti-TH antibody. Therefore, we concluded that the weak expression of EGFP is probably due to the silencing of the integrated transgene of prTH-EGFP-RAG-DsRed-IRESneo.

### 2.5. Exposure to TCDD Increased Neuronal and TH-Positive Cell Populations

**Figure 5:**
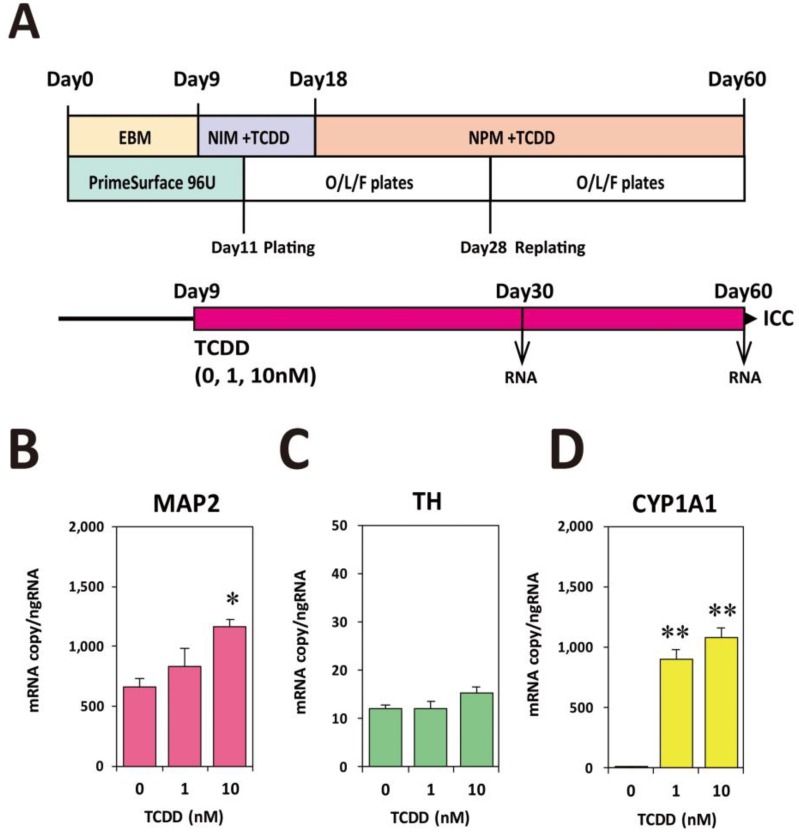
Exposure schedules of KhES1rTHEGFP and neuronal marker expression on Day30. (**A**) Schematic presentation of TCDD exposure. Cells were exposed to TCDD (0, 1, 10 nM) from Day9 to Day60. From Day9 to Day17, the NIM medium containing TCDD was changed with a fresh one every two days. From Day28 until the end of the experiments, the NPM medium containing TCDD was changed with a fresh one every three days. (**B**) RT-qPCR analysis of MAP2 mRNA expression. (**C**) RT-qPCR analysis of *TH* mRNA expression. (**D**) RT-qPCR analysis of CYP1A1 mRNA expression. Data are expressed as mean ± SE. One-way ANOVA was carried out followed by Dunnett’s test as post hoc (*, *p* < 0.05; **, *p* < 0.01).

We used the above-mentioned KhES1rTHEGFP cell line for EB formation and neural differentiation, which can be considered as a subline derived from KhES1 wild type, to examine the effects of TCDD exposure. We continuously added TCDD (0, 1, 10 nM) to the cultures from Day9 through Day60 (Figure 5A). RT-qPCR analysis carried out using total RNA collected on Day30 showed that the copy number of MAP2 mRNA significantly increased in a dose-dependent manner (Figure 5B). The *TH* mRNA copy number tended to increase, but the increase was not statistically significant (Figure 5C). For the confirmation of AHR activation, the levels of cytochrome P450 1A1 (CYP1A1) mRNA, which is a biomarker of dioxin exposure, were measured. As shown in Figure 5D, although a slight level of CYP1A1 mRNA expression was detected in the control group, remarkable inductions were detected in the 1 nM and 10 nM TCDD groups.

**Figure 6:**
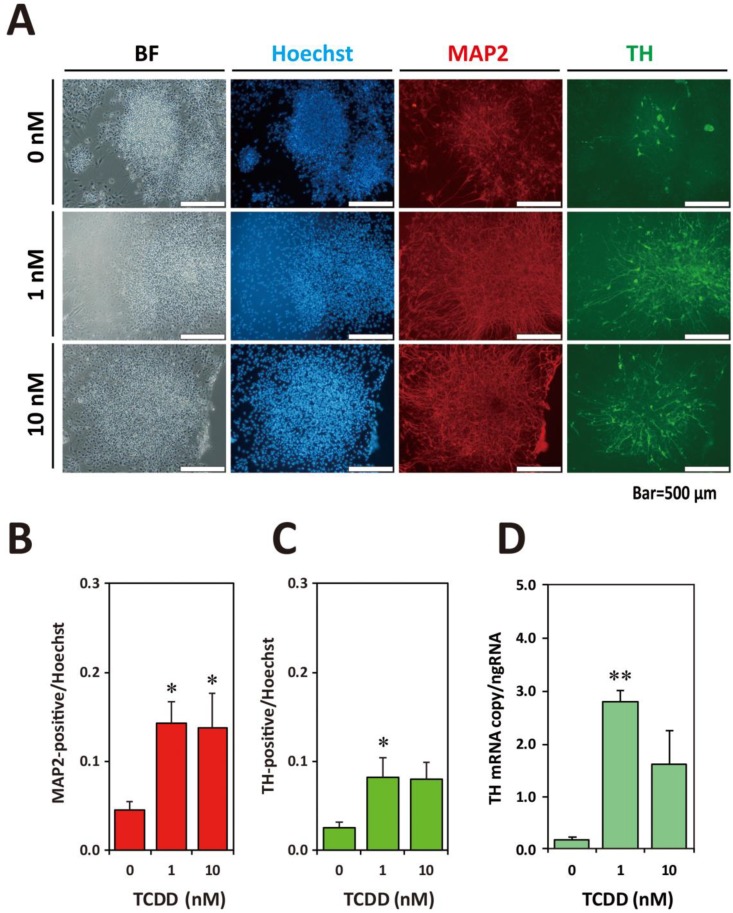
Effects of TCDD exposure on neural cell differentiations. (**A**) ICC on Day60 of the differentiated cells derived from KhES1rTHEGFP. During neuronal differentiation culture, the cells were exposed to TCDD (0, 1, and 10 nM) from Day9 to the end of culture (Figure 5A). BF, Bright-field; Hoechst, nucleus stained with Hoechst 33342; Red, MAP2 immunostaining; Green, TH-immunostaining. (**B**) MAP2-positive cell number. (**C**) TH-positive cell number. Data are expressed as mean ± SE (*N* = 5–6). (**D**) RT-qPCR analysis of *TH* mRNA expression levels. Data are expressed as mean ± SE (*N* = 3). One-way ANOVA was carried out followed by Dunnett’s test as post hoc (*, *p* < 0.05; **, *p* < 0.01).

Furthermore, ICC using the anti-MAP2 and anti-TH antibodies was carried out on Day60. The numbers of TH- and MAP2-positive cells were clearly increased in the 1 nM and 10 nM TCDD-exposed groups (Figure 6A). Image analysis of differentiated MAP2- and TH-positive cells indicated the statistically significant increases in the MAP2-positive cell number in the 1 nM and 10 nM TCDD-exposed groups (Figure 6B) and in the TH-positive cell number in the 1 nM TCDD-exposed group (Figure 6C). RT-qPCR analysis on Day60 showed that the copy number of *TH* mRNA significantly increased in the 1 nM TCDD-exposed group (Figure 6D). These results indicate that continuous exposure to TCDD stimulates the increase in neuronal cell population during developmental processes.

## 3. Discussion

In the present study, we employed the bulk-passage culture system to evaluate the effect of chemicals on neuronal cell differentiation from hESCs. We surprisingly found that TCDD exposure increases neuronal cell population, especially MAP2- and TH-positive cells. TH is a rate-limiting enzyme in dopamine synthesis [31,39]. Although the mechanism underlying this population increase is unclear, it is suggested that AHR activation signaling might positively stimulate the differentiation of neurons. The effects of TCDD toxicity on the development of the central nervous system have not been well studied. In general, neural rosettes are observed during the developmental stage of neural progenitors in stem cell culture [40]. Our study showed that in the early culture stage for neural initiation (Day9), the neural rosette number was significantly increased by short-term TCDD exposure. This fact suggests that neuronal stem cells in the EB stage are more sensitive to dioxin exposure than ESCs or matured neural cells.

The expression level of TCDD biomarker CYP1A1 has been measured in other several similar examinations using hESCs. In such preliminary experiments, we found an interesting phenomenon that CYP1A1 induction is not induced in the undifferentiated hESCs and differentiated neuronal cells, but in EB on PrimeSurface U96 and out-growing cells from attached EB on O/L/F plates. These unique expression and induction profiles of the CYP1A1 gene must be due to epigenetic modification differences in each cell type during neuronal differentiation. We are now conducting epigenetic analysis of its molecular mechanism. We will report these precise data in detail in our next paper (unpublished data).

The neurotoxic effects of TCDD have been analyzed over the last three decades, mostly by the developmental and behavioral analyses of animals during gestational exposure [41]. Fetal exposure to TCDD in rats and mice was shown to induce various effects on brain development. The function of GABAergic neurons in the preoptic area was shown to be impaired in rats [42]. The maturation of granule neuron precursors was reported to be disturbed in the mouse cerebellum [43]. It has been shown that Ahr is a primary target molecule because no decrease in the spatial learning ability nor any abnormality of hippocampal nervous system cells was found in *Ahr* knockout mice [44]. Moreover, it is known to produce neocortical dysgenesis through the activation of p27Kip1 by TCDD-liganded Ahr (TCDD-Ahr) in the murine fetal period [45]. Human neuronal progenitor cells (hNPCs) were reported to be not responsive to TCDD owing to a lack of functional AHR [46], whereas growth arrest from G1 to S phase was shown to occur in an experiment in which p27Kip1 and cyclinD1 expression levels were affected by TCDD in human neuronal progenitor cells [47]. Thus, studies using neural progenitor or precursor cells have not yet given consistent conclusions regarding AHR activation. Our data are also quite different from their data using hNPCs described above. The discrepancy may be due to differences in the nature of their neural progenitor cells and neuronal cells directly differentiated from the hESCs we used.

We have already examined if TCDD affects the proliferation of hESC-derived cells using KhES3 cell line in a preliminary experiment. The experiment was done in parallel with our previous study of methylmercury neurotoxicity [29]. Cells out-growing from EB just after plating on O/L/F-coated plates on Day11 were exposed to increasing concentrations of TCDD. From the day of plating and for 13 days, MTT assay was performed every day to examine the viability of differentiating cells. However, even at the highest concentration of 1000 nM, the cells were resistant to continuous exposure to TCDD for 13 days (Figure S3, Supplementary Materials). Taken together, the numerous studies described above and our preliminary data indicate that the mechanisms by which environmental dioxin exposure exerts its effect on brain functions in humans are still unclear. The present study is the first to show that TCDD affects the differentiation of the neuronal cells derived from human ESCs. The EB stage is equivalent to a very early stage of human embryogenesis and may contain naïve neuroprogenitors sensitive to dioxin.

Our study showed an inducible effect of TCDD on the differentiation of TH-positive neuronal cells. The effects of TCDD-AHR signaling on the *TH* gene and TH-positive cell development were seen in the literature [48,49,50]. The overexpression of Ahr cDNA in the murine neuroblastoma Neuro2a cell induced *Th* expression [48]. A regulatory sequence called Ahr responsive element III (AHRE-III) was identified within the *Th* gene from the -285 to -167 upstream region [49]. The upregulation of *Th* gene expression by AHRE-III was evidenced by Neuro2a cell-based reporter assay and TCDD exposure. Furthermore, by using AHRE-III-driven luciferase transgenic mice, the authors revealed that fetal exposure to TCDD upregulated *Th* expression and also induced overgrowth of Th-positive neurons in the midbrain [50]. This finding suggests that TCDD-AHR signaling during fetal development enhances Th-positive cell proliferation. The overgrowth of Th-positive neurons induced by TCDD in an animal model indicated the possible involvement of Ahr signaling in the etiology of neurodevelopmental disorders through dopaminergic neuronal dysfunction [50]. The results from our present study are consistent with their results showing that TCDD-Ahr signaling in the fetal stage increases the number of Th-positive neurons [50].

A combination of L-DOPA and Carbidopa has been used as a treatment for Parkinson’s disease (PD) [51]. Carbidopa is an analogue of L-DOPA with a methyl group attached to the α-carbon and a hydrazine moiety. Although Carbidopa itself has no therapeutic effect, it is known to enhance the efficacy of L-DOPA. Most recently, Carbidopa was found to be an Ahr ligand and to induce Ahr nuclear translocation and CYP1A1 as an agonist [52]. Although the relationship between dopamine neuron activation and Ahr signaling is unknown, it is important to examine the effects of endogenous ligands of AHR (e.g., tryptophan metabolites such as 5,11-dihydroindolo[3,2-b]carbazole-6-carboxaldehyde (FICZ) [53] and Kynurenine [54]). 

Since our present study is only a description of the phenomenon, we have not examined which genes downstream of the AHR signal are involved in the growth of neural cells. In the stem cell-derived three-dimensional culture system, an EB differentiates into the so-called organoid [55,56]. For example, the stem cell-derived three-dimensional culture system produced a cerebral-like architecture (cerebral organoid) that mimicked the complexity of the human brain [57]. The bulk-passage culture protocol in this study is not aimed at organoid formation; however, a three-dimensional structure in the culture well still has characteristics of EB containing naïve differentiating cells. It is difficult to introduce cDNA or siRNA into all cells constituting EB having organoid-like properties. Therefore, there are many technical issues to be overcome in elucidating the mechanism of genes downstream of AHR.

As an in vitro toxicity test system alternative to the animal experiment [27,28], the generation of hESCs that allow live imaging of the development of TH-positive dopaminergic neurons was very important for our project funded by the Japanese government (Ministry of Health, Labor and Welfare). As part of the project, we attempted to develop a Pluripotent Stem Cell Battery, which will enable the monitoring of the differentiation process that is highly similar to the differentiation of three germ layers and further organogenesis. The expected Pluripotent Stem Cell Battery tests one particular chemical by aligning various pluripotent stem cells (ES/iPS cells) generated from several animal species. By comparing the species differences between human stem cell data and those of animals, it would be possible to predict human susceptibility to developmental toxicity based on the in vivo toxicity data of an animal from which stem cells are derived. The expected Pluripotent Stem Cell Battery will be a breakthrough for the rapid and precise investigation of human susceptibility or side effects to toxic chemicals or drugs, as well as for reducing the number of experimental animals used [58].

To establish a more convenient system, we tried to generate transgenic human ESC lines that had the *TH*-driven EGFP reporter gene for applications in live imaging. The live imaging of dopaminergic neurons driven by the *TH* promoter provides a simple and useful system to monitor dynamic processes of TH-positive cell development [39]. The rat promoter DNA used was very reliable for stable transfection studies [39,59]. Many reasons for our lack of success can be speculated. One reason is that two rat promoter sequences (i.e., the *Th* and *Actb* genes) were silenced by epigenetic modifications in ESCs [60]. Otherwise, human transcription factors for the activation of these genes may have insufficient activity for these rat promoters. Rather than simple transfection, it is necessary to use human *TH*-promoter DNA with knockin vector or the gene editing technology [61].

Previously, in collaboration with Dr. Sone Hideko’s group at Yokohama Pharmaceutical University, we reported human ESC experiments on neurodevelopmental toxicity of methylmercury [29]. In the research, not only the number of cells but also the neurite length and the number of branches were automatically measured using an IN Cell Analyzer 2000 (GE Healthcare UK Ltd., Amersham, Buckinghamshire, UK). However, in mechanical measurements that focus on cell morphology, including time-lapse imaging devices [62], the cell density must be reduced because it must be observed with a small number of cells. In our bulk-passage culture system, the cell density was maintained high. Therefore, the neurite length and the number of branches could not be measured in this study. Perhaps ESCs having the *TH*-driven EGFP transgene will be a useful tool in solving such difficulties. Although we were unable to produce *TH*-driven EGFP reporter transgenic ESCs in this study, further attempts to generate such genetically modified ES/iPS cells are necessary to understand the underlying mechanisms of organogenesis of the human central nervous system as well as to establish a new in vitro toxicity test system. 

## 4. Materials and Methods 

### 4.1. Reagents and Plastic Wares

Dulbecco’s Modified Eagle’s Medium (DMEM) (1×) without phenol red, DMEM/F-12 (1×) liquid 1:1 without phenol red, DPBS(-), Neurobasal^®^ Medium (1×) without phenol red, Knockout™ Serum Replacement (KSR), N-2 Supplement (100×), B-27^®^ supplement minus vitamin A (50×), collagenase type IV, recombinant human brain-derived neurotrophic factor (BDNF), GlutaMAX™-I (100×), Non-Essential Amino Acids Solution (NEAA, 10 mM, 100×), TrypLE™ Select, penicillin -streptomycin (P/S, 5000 units/mL, 5000 µg/mL), 2-mercaptoethanol (2ME, 55 mM in D-PBS, 1000×), Lipofectamine 2000, Lipofectamine LTX, Alexa Fluor 488 F(ab’)2 fragment of goat anti-rabbit IgG (H+L), Alexa Fluor 568 goat anti-mouse IgG (H+L) as the secondary antibody, and Alexa Flour 568 rabbit anti-mouse IgG were purchased from Invitrogen Corporation (Carlsbad, CA, USA). Gelatin from porcine skin type A, Poly-L-ornithine solution 0.01%, laminin from Engelbreth-Holm-Swarm murine sarcoma basement membrane, and the mouse monoclonal anti-MAP2 antibody (clone HM-2) were purchased from Sigma-Aldrich (St. Louis, MO, USA). Fetal bovine serum (FBS) HyClone was obtained from GE Healthcare UK Ltd. (Little Chalfont, UK). Human fibronectin protein and Accutase cell detachment solution were obtained from R&D Systems Inc. (Minneapolis, MN, USA). The rabbit polyclonal antibody to TH was obtained from Millipore (Billerica, MA, USA). Mitomycin C, recombinant human basic-FGF (bFGF), all-trans retinoic acid (RA), and the ROCK inhibitor Y-27632 were purchased from Wako Pure Chemical Industries (Chuo-ku, Osaka, Japan). Hoechst 33342 solution was purchased from Dojindo Laboratories (Kamimashiki-gun, Kumamoto, Japan). 2,3,7,8-Tetrachloro-*p*-dioxin (TCDD, purity >99.5%, solution in dimethyl sulfoxide (DMSO)) was purchased from Cambridge Isotope Laboratory (Andover, MA, USA). DMSO and MTT cell-counting kits were purchased from Nacalai Tesque, Inc. (Nakagyo-ku, Kyoto, Japan). An RNeasy Mini Kit was purchased from QIAGEN (Hilden, Germany). LightCycler^®^ 480 SYBR Green I Master was purchased from Roche Diagnostics GmbH (Mannheim, Germany). The PrimeScript^®^ RT reagent Kit and TaKaRa Ex-Taq were purchased from Takara BIO Inc. (Otsu, Shiga, Japan). All oligonucleotides purified by gel filtration were purchased from Hokkaido System Science (Sapporo, Hokkaido, Japan). Restriction enzymes were purchased from New England Biolabs Inc., (Ipswich, MA, USA). The antibiotic G-418 sulfate and pGL3-basic vector were obtained from Promega (Madison, WI, USA). Nerve growth factor (NGF) was obtained from Collaborative Biochemical Products Inc., (Bedford, MA, USA). Pfu Turbo DNA polymerase and pMC1neo-polyA were obtained from Stratagene (La Jolla, CA, USA). The plasmid pRetroQ-DsRed-monomer-N1 vector was obtained from Clontech Laboratories (Mountain View, CA, USA). Culture dishes (60 mm) and 24-well plates were obtained from Corning Costar (Cambridge, MA, USA), and PrimeSurface 96U plates were obtained from Sumitomo Bakelite Co., Ltd., (Shinagawa-ku, Tokyo, Japan). Countess™ Cell Counting Chamber Slides were obtained from Thermo Fisher Scientific (Minato-ku, Tokyo, Japan).

### 4.2. Feeder Cell Culture and Miscellaneous

Mouse embryonic fibroblasts (MEFs) were prepared in our laboratory as previously described [29]. The SNL 76/7 (hereafter SNL) cell line was obtained from RIKEN Bio Resource Center (Tsukuba, Ibaraki, Japan). MEFs and SNL were routinely grown in 0.1% gelatin-coated 60 mm culture dishes containing DMEM, supplemented with 10% FBS at 37 °C in a humidified atmosphere of 5% CO_2_ and 95% air. HepG2, PC12, and SK-N-SH cell lines were also obtained from RIKEN. HepG2 cells were cultured in DMEM containing 10% FBS and P/S. Undifferentiated PC12 and SK-N-SH cells were maintained in DMEM containing 10% FBS, 2 mM L-glutamine, and P/S. NGF (100 ng/mL) was added to induce the differentiation of neuronal cells [63,64].

### 4.3. Human ESC Maintenance Culture

Human ESCs (KhES1 and KhES3) were provided by the Institute for Frontier Medical Science (Kyoto University, Kyoto, Japan). All experiments using hESCs were approved by the Ethics Committee for Human ES Cell Usage of the University of Tokyo, in accordance with the guidelines of the Ministry of Education, Culture, Sports, Science, and Technology (MEXT) of Japan. The hESCs were maintained as described previously [29]. First, MEF or SNL cells as the feeder cells were plated on gelatin-coated 60 mm dishes at 9.5 × 10^3^ cells/cm^2^ and 3.8 × 10^3^ cells/cm^2^, respectively. The MEF and SNL cells were mitotically inactivated by treating them with 5  µg/mL mitomycin C for 2 h. Then, human ESCs were plated on the feeder cells in the embryonic stem cell medium (ESM) composed of DMEM/F12 containing 20% KSR, 1× NEAA, 2 mM L-glutamine, 73.5 nM 2 ME (Sigma-Aldrich), and 5 ng/mL bFGF (added by one pipetting immediately after changing ESM with a fresh one minus bFGF). The medium was replaced with a fresh medium every day. After ESC colonies were formed, which grow fast, hESCs were dissociated from the dishes using TrypLE™ Select and passaged. A typical hESC colony is presented in Figure 1.

### 4.4. O/L/F-Coated Plates

To form an extracellular matrix, ornithine/laminin/fibronectin (O/L/F)-coated plates were prepared as follows. Poly-L-ornithine (0.01%) was added to a 24-well plate (0.5 mL/well) and a 6-well plate (1 mL/well), and the plates were incubated at RT overnight. After washing with PBS three times, a mixture of laminin (20 mg/mL) and fibronectin (5 mg/mL) was added to the 24-well plate (0.5 mL/well) and the 6-well plate (1 mL/well), and then incubated overnight for cells to adhere onto the plate surface. Just before plating the cells, the supernatant was obtained by aspiration.

### 4.5. Neural Differentiation Culture

Figure 1A shows a schematic diagram of bulk-passage culture for neuronal differentiation from hESCs. For the generation of EBs, the hESCs maintained in MEFs were treated with TrypLE™ Select at 37 °C and checked at 1 min intervals for the detachment of MEFs from the plate and the formation of cell masses from the hESC colony. The detached MEFs and the masses of hESCs were settled at a unit gravity in a 15 mL tube with 10 mL EB medium to correct hESC masses from settling only in the bottom of the tube. The purified hESCs were separated into single cells by gentle pipetting. The cells were seeded on a PrimeSurface 96U plate at 9.0 × 10^3^ cells/well in the embryoid body medium (EBM) consisting of DMEM/F12 containing 20% KSR, 1× NEAA, 2 mM L-glutamine, 73.5 nM 2-ME (Sigma-Aldrich), and 10 µM ROCK inhibitor Y-27632 [65]. The start of the day of culture on PrimeSurface 96U was designated as Day0. From Day0 to Day8, EBM was changed with a fresh one every two days. A typical hESC-derived EB is presented in Figure 1B. On Day9, after discarding half of the medium, a fresh neural initiating medium (NIM) consisting of DMEM/F12 plus Neurobasal^®^ Medium (1:1), containing 1× N-2 Supplement, 1× B-27^®^ Supplement, 1× GlutaMAX™-I, and P/S was added. From Day9 to Day17, the NIM medium was changed with a fresh one every two days. On Day11 of culture, the EBs were transferred to O/L/F-coated 24-well plates at 2 EBs/well and cultured in NIM to initiate neural differentiation. On Day18, the medium was changed with neural proliferation medium (NPM) consisting of DMEM/F12 plus Neurobasal^®^ Medium (1:1), 2× N-2 Supplement, 2× B-27^®^ Supplement, 1× GlutaMAX™-I, P/S, and 20 ng/mL bFGF. On Day28, the entire differentiated cell mass present in one well was detached using Accutase and transferred to one well of O/L/F-coated 12-well and 6-well plates. From Day28 until the end of the experiments, the NPM medium was changed with a fresh one every three days. The typical features of differentiated neuronal cells on Day40 are presented in Figure 1B.

### 4.6. Quantitative RT-PCR Analysis

Total RNA from hESC derivatives was collected with an RNeasy Mini Kit from three wells. Gene expression levels in the hESC derivatives were investigated by quantitative RT-PCR (RT-qPCR) using a high-throughput real-time thermal cycler (Light Cycler 480 system, Roche, Basel, Switzerland). The PCR conditions were as follows: 95 °C for 5 min, 45 cycles at 95 °C for 10 s, 60 °C for 10 s, and 72 °C for 20 s. The primers used in this study are shown in Table S1 (Supplementary Materials). Quantitative data were obtained by calculating the absolute copy number as previously described [66].

### 4.7. Transfection and Stably Transformed Cell Cloning

The plasmid prTH-EGFP, which has a functional 10-kb 5′-flanking region of the rat tyrosine hydroxylase (*Th*) gene and the EGFP reporter, was kindly provided by Dr. Okano of Keio University [32,35,59]. First, the rat β-actin gene (RAG) promoter was cloned from the Sprague Dawley rat genome by PCR using the forward primer (5′-AGA GAT CTA CCT CTT CCT CAA CTC ACT TCT CTC TA-3′) and the reverse primer (5′-TCT TCC ATG GCG AAC TAT CAA GGC ACA AAA GAG GG-3′) and then subcloned into the pGL3-basic vector. Promoter activity was determined by Luciferase assay. The DsRed open reading frame was isolated from the pRetroQ-DsRed-Monomer-N1 vector and connected with RAG. The PCR amplified IRES sequence [66] was inserted into the pMC1neo-polyA plasmid, and then the RAG-DsRed fragment was connected to the upper region of the IRES-neo cassette, resulting in the plasmid pMC1-RAG-DsRed-IRESneo. The rat *Th* promoter and the EGFP fragment isolated from prTH-EGFP were inserted into the pMC1-RAG-DsRed-IRESneo plasmid to generate the construct prTH-EGFP-RAG-DsRed-IRESneo (Figure S2A, Supplementary Materials). The construct was first transfected to PC12- and SK-N-SH- derived differentiated neuronal cells using lipofectamine 2000 to check the rat *Th* promoter activity. The *Not* I restriction enzyme-linearized prTH-EGFP-RAG-DsRed-IRESneo DNA was then transfected to KhES1 cells by lipofectamine LTX to generate stable transformants of human ES cells. Some DsRed-positive colonies cultured with SNL feeder cells in the presence of G418 were selected and cloned.

### 4.8. Immunocytochemistry and Image Analysis

Double immunostaining for TH and MAP2 was performed at different stages of neuronal differentiation in our experiment. Stem cells were fixed for 10 min with 4% paraformaldehyde. Before staining, the cells were permeabilized in 0.1% Triton X-100 in PBS. They were incubated with 1% BSA blocking solution, followed by overnight incubation with a mixture of primary antibodies specific for TH and MAP2 at 1:1000 dilution in blocking solution. To remove the unbound primary antibody, the cells were washed three times with PBS and subsequently incubated for 1 h at room temperature with a mixture of the Alexa 568-labeled goat anti-rabbit IgG secondary antibody and the anti-mouse IgG secondary antibody at 1:1000 dilutions in blocking solution. The cells were washed three times with PBS, and the nuclei were stained with 2 µg/mL Hoechst 33342 for 15 min at room temperature. Microscope images were taken from three wells. All values in this study were presented by the number of ratio of the target cells to the Hoechst-stained cells in the same image. Images were analyzed using ImageJ software (Version 1.52a, National Institutes of Health, Bethesda, MD, USA).

### 4.9. Statistical Analysis

Statistical analysis of the number of rosettes formed, gene expression level, and TH-positive cell count was performed using KakeidaGraph Version 4 for Windows (Synergy Software, Reading, PA, USA). All data were expressed as means ± SE. One-way ANOVA was carried out followed by Dunnett’s test as post hoc. Statistical significance was indicated by *p*-value (*p* < 0.05).

## 5. Conclusions

The bulk-passage system used in this study enabled the observation of the ratios of several types of differentiating cells. We found that embryonic cells at the EB stage and neuronal progenitors are quite resistant to TCDD exposure. Interestingly, we found that AHR activation in the early embryonic stage, which is expected to be identical to the EB stage, may be involved in the induction of neural differentiation. In particular, the TH-positive cell population was increased by TCDD. AHR endogenous ligands, such as FICZ, may play important roles in neurons and the central nervous system. In this study, we were unable to establish *TH*-promoter-driven EGFP human ESC lines. We are trying to establish cell lines that are applicable to conventional live-imaging systems for the embryonic stem cell battery.

## Supplementary Materials

Supplementary materials can be found at https://www.mdpi.com/1422-0067/20/11/2687/s1.

## Abbreviations

hESC	human embryonic stem cell
EST	embryonic stem cell test
EB	embryoid body
MEF	mouse embryonic fibroblast
TCDD	2,3,7,8-tetrachlorodibenzo-*p*-dioxin
AHR	aryl hydrocarbon receptor
MAP2	microtubule-associated protein 2
TH	thyroxine hydroxylase
ESM	embryonic stem cell medium
EBM	embryoid body medium
NIM	neuronal induction medium
NPM	neural proliferation medium
ROCK	Rho-associated coiled-coil forming kinase
O/L/F	ornithine/laminin/fibronectin
RAG	rat β-actin gene
EGFP	enhanced green fluorescent protein
DsRed	discosoma red fluorescent protein
IRES	internal ribosome entry site
ICC	immunocytochemistry
RT-qPCR	reverse transcription quantitative polymerase chain reaction
ANOVA	analysis of variance
