# Supplementary material for: Tyroxine Hydroxylase-Positive Neuronal Cell Population is Increased by Temporal Dioxin Exposure at Early Stage of Differentiation from Human Embryonic Stem Cells
Source: Int J Mol Sci. 2019 May 31;20(11):2687. doi: 10.3390/ijms20112687 (PMC6600215; doi:10.3390/ijms20112687)

Supplementary Table S1. Primers used in this study

Gene	Forward (5' → 3')	Reverse (5' → 3')	Size	Reference
NES	AGCGTTGGAACAGAGGTTG	AGGCTGAGGGACATCTTGA	171 bp	NM_006617
MAP2	GGAGTAACCAAGAGCCCAGA	CTCTGCGAATTGGCTCTGAC	166 bp	NM_002374
TH	GAGGCCATCATGGTAAGAGG	CTTCTCCTCAAAGGCCACAG	243 bp	NM_199292
SOX17	GAACGCTTTCATGGTGTGGG	TCTGCCTCCTCCACGAAG	150 bp	NM_022454
FOXA2	ATGCACTCGGCTTCCAGTAT	CACGTACGACGACATGTTCAT	225 bp	NM_021784
KDR	CAGGATGCAGAGCAAGGTG	TCAAGAGAAACACTAGGCAAACC	93 bp	NM_006206
AHR	AGAGTTGGACCGTTTGGCTA	AGTTATCCTGGCCTCCGTTT	167 bp	NM_001621
CYP1A1	CCAGGCTCCAAGAGTCCACC	GCCTTTGGGGACCTGAGG	183 bp	BC023019
ACTB	TCGTCGTCGACAACGGCT	CGTGCTCGATGGGGTACTTC	198 bp	NM_001101

Supplementary Figure S1

Sarma et al., *Int J Mol Sci*

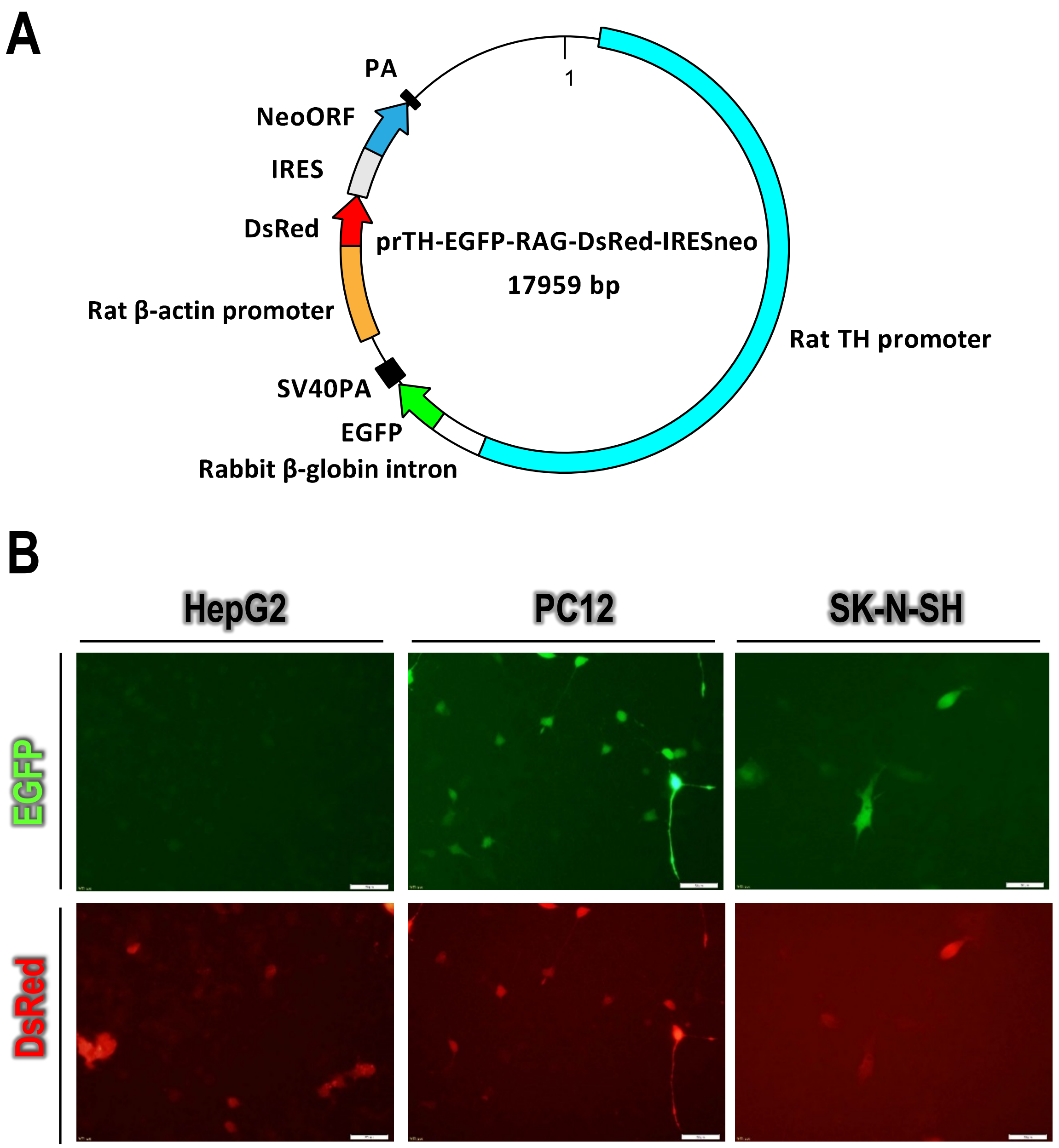

Supplementary Figure S2

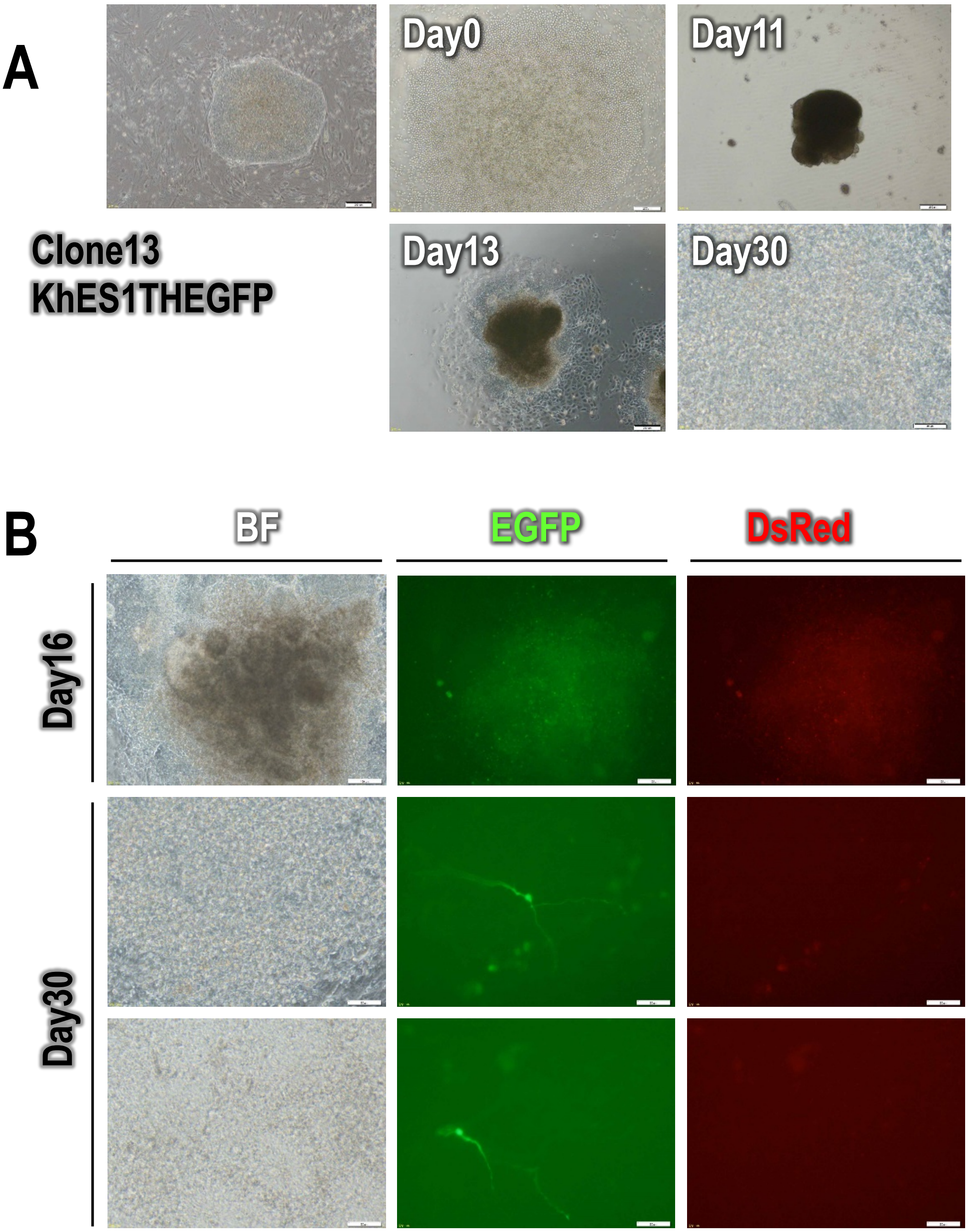

Supplementary Figure S3

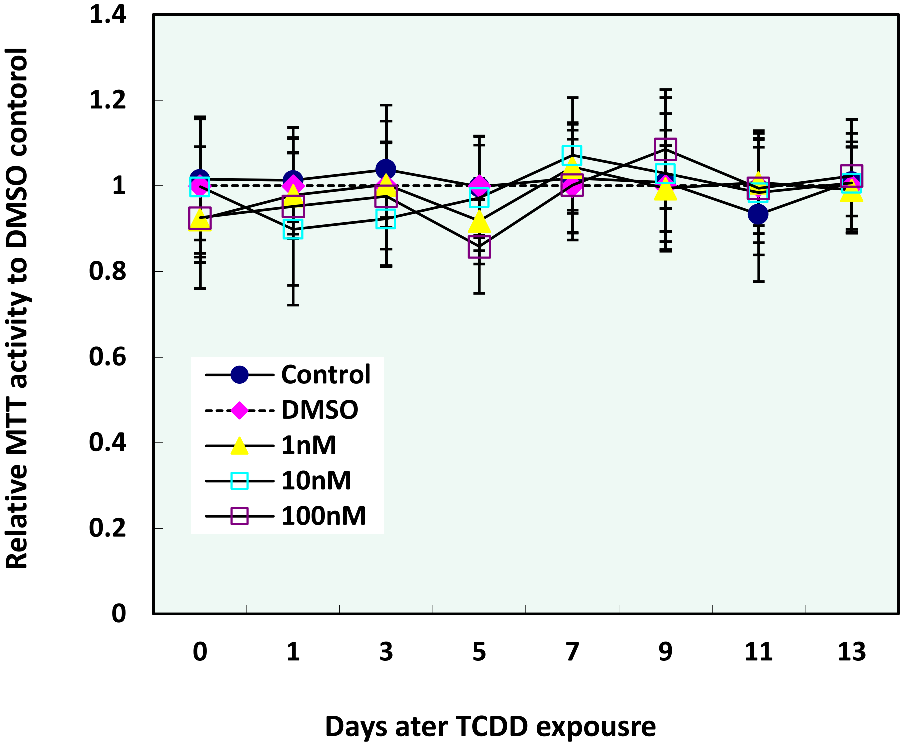

Supplement: Supplementary file 1 [file ijms-20-02687-s001.pdf]
